# Autistic people outperform neurotypicals in a cartoon version of the Reading the Mind in the Eyes

**DOI:** 10.1002/aur.2782

**Published:** 2022-07-20

**Authors:** Liam Cross, Andrea Piovesan, Gray Atherton

**Affiliations:** ^1^ Department of Psychology Edge Hill University Liverpool UK; ^2^ Department of Design and Planning in Complex Environments, Università Iuav di Venezia Venice Italy

**Keywords:** anthropomorphism, autism, cartoon, emotion recognition, Reading the Mind in the Eyes

## Abstract

Prior research suggests that while autistic people may demonstrate poorer facial emotion recognition when stimuli are human, these differences lessen when stimuli are anthropomorphic. To investigate this further, this work explores emotion recognition in autistic and neurotypical adults (*n* = 196). Groups were compared on a standard and a cartoon version of the Reading the Mind in the Eyes test. Results indicated that autistic individuals were not significantly different from neurotypicals on the standard version. However, autistic people outperformed neurotypicals on the cartoon version. The implications for these findings regarding emotion recognition deficits and the social motivation account of autism are discussed and support the view of socio‐cognitive differences rather than deficits in this population.

**Lay Summary:**

The Reading the Mind in the Eyes test and a cartoon version were tested on autistic and neurotypical adults. Autistic adults were not significantly different on the original test compared to neurotypicals, but they outperformed neurotypical adults on the cartoon version.

## INTRODUCTION

Autistic people are often described as mindblind or having poorer socio‐cognitive skills than neurotypicals (NTs) (Baron‐Cohen, 1997). The mindblind account not only pathologises the autistic condition (Duffy & Dorner, 2011), but it also fails to consider the double empathy problem (Milton, 2012), which occurs when NTs do not consider the autistic person's perspective. Failures in double empathy are often not considered failures, as neurotypical ways of relating to the world are used as benchmarks by which neurodivergent preferences or strategies can be measured (Pellicano & den Houting, 2021). Many socio‐cognitive tests in general, but facial emotion recognition (FER) tests in particular, reflect the double empathy problem in autism research. FER assessments are often explicitly designed to exploit NT strengths and autistic deficits. For instance, the Reading the Mind in the Eyes test (RME) (Baron‐Cohen et al., 2001) was devised to identify areas of autistic differences, such as irregular eye‐gaze (Tanaka & Sung, 2016). While understanding deficits can help identify areas of need, there is a growing movement to capture the strengths inherent to neurodiverse conditions such as autism. In this way, tests such as the RME may themselves be changed to reveal autistic strengths and understand the contexts in which differences between autistic and NT people are lessened.

Research suggests that one area of social‐cognitive strength in autistic people centers upon anthropomorphism (Atherton & Cross, 2018). Anthropomorphism refers to social cognition about nonhuman agents and is commonly deployed when interacting with animals, robots, dolls, cartoons, or avatars (Yamada et al., 2013). As evidenced by the uncanny valley effect (Mori et al., 2012), NTs are better at relating to real humans than anthropomorphic stimuli (Cheetham et al., 2014). However, autistic adolescents have been found to have improved FER using an anthropomorphised version of the Karolinska directed emotional faces task (Cross et al., 2019). NTs also regularly perform more poorly on FER tasks when stimuli are anthropomorphic rather than human (Atherton & Cross, 2018, 2019). This likely stems from their expertise and familiarity with similar types of human agents repeatedly encountered through everyday life (Cheetham et al., 2014). In contrast, there is a good deal of evidence that autistic people do not show this bias for the purely human. Unlike NTs, autistic people do not detect uncanniness in response to anthropomorphic faces (Feng et al., 2018) or voices (Kuriki et al., 2016). Performing FER tasks on anthropomorphic agents does not appear to disadvantage autistic people as they do NTs. For instance, Davidson et al. (2019) found that autistic children were less able to perform human FER compared to NTs, but groups did not differ on canine FER. Whyte et al. (2016) found that autistic adolescents experienced hypoactivation across the face‐processing neural network when performing FER tasks with human faces but were indistinguishable from NTs when processing animal faces. The autistic group also showed greater activation in the affective regions of the FER network in response to the animal faces. Brosnan et al. (2015) tested autistic and NT adolescents on FER with cartoon and human faces. While there was a human FER advantage for NTs, autistic participants outperformed NTs on cartoon FER.

A penchant for anthropomorphic rather than human social stimuli is well documented in the autistic community (for a review, see Atherton & Cross, 2018). Animals and cartoons consistently appear in studies that document and categorize autistic people's restricted interests (RIs) (Cho et al., 2017; Klin et al., 2007; Nowell et al., 2021; South et al., 2005), a common feature of the autistic phenotype that may help explain social differences in the population (Carter et al., 2020). While time spent engaging with any RI is enjoyable (Mercier et al., 2000), engagement with anthropomorphic RIs may also support ToM development, as reported by autistic adolescents (Atherton et al., 2018) and their parents (Rozema, 2015). Cartoons, for instance, exaggerate the human aspects of a nonhuman agent, which may increase empathy for the agent and boost understanding of the artist's intent (Carbajal‐Carrera & Sanchez‐Castro, 2020). Cartoon media is also appreciated by NTs, and may not represent an ‘unusual’ interest that could differentiate autistic and NT people (Nowell et al., 2021). Ease in deciphering FER in cartoons and a shared appreciation by autistic and NT people alike suggest cartoons could be a way to explore FER strengths in the autistic population.

In sum, while autistic individuals and those who score high on autistic traits usually perform more poorly on FER tasks such as the RME, using an anthropomorphic version of this task may reduce these differences. A recent study by Atherton and Cross (2021) developed a cartoon version of the RME, and results showed that those high in autistic traits did not perform at a deficit compared to those with lower levels of autistic traits. Here we aim to replicate and extend these findings with an autistic sample. We hypothesized that autistic people would correctly identify fewer items on the RME than NTs, but this would not be the case for the cartoon version of the RME (C‐RME). That is, there would be no difference in the scores of those with autism spectrum condition (ASC) and neurotypicals (NTs) in the cartoon version of the RME.

## METHODS

**FIGURE 1:**
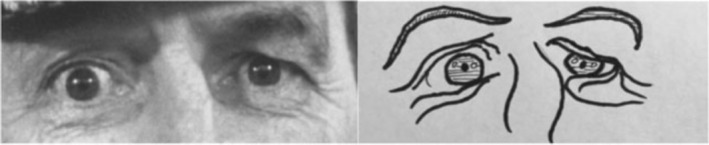
Practices item for the adapted Reading the Mind in the Eyes (RME) based on the original Baron‐Cohen et al. (2001) RME. The correct emotion is panicked, presented with the following three foils: jealous, arrogant, and hateful

A 2 × 2 between groups methodology was employed, where individuals diagnosed with autism and NTs took either the RME or the C‐RME online. Comparison items for the two measures can be found in Figure 1.

Individuals first reported basic demographic information and then participated in either the RME or the C‐RME. Individuals were shown 36 pictures (RME) or drawings (C‐RME) of eyes and asked to pick which emotion the eyes portrayed out of four options (one correct, three foils). These pictures were presented one at a time with the order randomized. To avoid issues with verbal comprehension and the RME (Peterson & Miller, 2012), participants were encouraged to hover their cursor over unfamiliar words that superimposed the Oxford dictionary definition and a sentence using the word. Participants then reported how difficult the emotion recognition test was on a continuum ranging from 0 (not at all difficult) to 100 (very difficult). This study took approximately 30 min to complete, all participants gave full informed consent, and those who chose to provide an email address entered a draw to win a £50 Amazon gift card. This study was reviewed and approved by the University of Wolverhampton's Ethics Review Board.

Individuals with a clinical diagnosis of autism were recruited via specific online autism forums and social media pages (Facebook, Reddit, and Wrong Planet) to participate online via Qualtrics. A sample of 100 autistic individuals (50 per condition) was sought. Power analysis using GPower indicated that a sample of 50 per condition afforded above 90% power to detect medium effect sizes, as seen in Atherton and Cross (2021). The survey was left live until the target sample size was reached. As a confirmation that participants had a clinical diagnosis, participants were asked, following their endorsement of a diagnosis, whether this diagnosis was received from a medical professional and at what age they had been diagnosed (Baron‐Cohen et al., 2015). Anyone who responded that they were self‐diagnosed with autism, meaning they had not received a clinical diagnosis from a medical professional, was excluded (*n* = 2). While this method is effective in confirming clinical diagnosis of ASC in online research (Baron‐Cohen et al., 2015), it should be noted that the study relied on such identification and did not clinically confirm the diagnosis of participants. Similarly, no data on symptomology, comorbidity or intelligence levels were collected and females were over represented in our sample. For these reasons, the authors wish to add a note of caution in interpreting and generalizing these findings.

Therefore, the final sample consisted of 98 autistic and 98 aged‐matched NT participants. Table 1 shows participants' demographics divided by the four experimental groups (NT in the RME condition, NT in the C‐RME condition, ASC in the RME condition, and ASC in the C‐RME condition).

**TABLE 1:** Participants' demographics divided by the four experimental groups.

Variable		NT		ASC		Total
		RME	C‐RME	RME	C‐RME	
*N*		50	48	50	48	196
Age mean (SD)		29.20 (10.74)	30.31 (10.95)	30.96 (12.97)	30.56 (14.83)	30.26 (12.39)
Sex (%)	Male	4 (8.0)	12 (25.0)	21 (42.0)	18 (37.5)	55 (28.1)
	Female	46 (92.0)	36 (75.0)	29 (58.0)	30 (62.5)	141 (71.9)
Ethnicity (%)	White	31 (62.0)	26 (54.2)	43 (86.0)	41 (85.4)	141 (71.9)
	Asian	13 (26.0)	14 (29.2)	1 (2.0)	0 (0.0)	28 (14.3)
	Hispanic	0 (0.0)	0 (0.0)	1 (2.0)	1 (2.1)	2 (1.0)
	Black	2 (4.0)	2 (4.2)	1 (2.0)	1 (2.1)	6 (3.1)
	Other	4 (8.0)	6 (12.5)	4 (8.0)	5 (10.4)	19 (9.7)

*Note*: *p < 0.05; **p < 0.01; ***p < 0.001.

## RESULTS

We compared the proportion of correct emotion recognition across the four experimental groups. First, outliers were identified within each experimental group by creating boxplots of the percentage of correct answers (boxplots are reported in Appendix S1). This exclusion criterion was set a priori in line with recommendations by Cousineau and Chartier (2010) and Meyvis and Van Osselaer (2018). Explorations of the boxplots revealed six outliers (four NTs in the RME condition and two ASCs in the C‐RME condition), which were excluded from analysis, in line with other work which tested an online version of the RME (Olderbak et al., 2015). A univariate ANOVA was then performed with the proportion of correct emotion recognitions as the dependent variable and Presentation Format (C‐RME vs. RME, random factor) and Diagnosis (autistic vs. NT, fixed factor) as the independent/subject variables.

**FIGURE 2:**
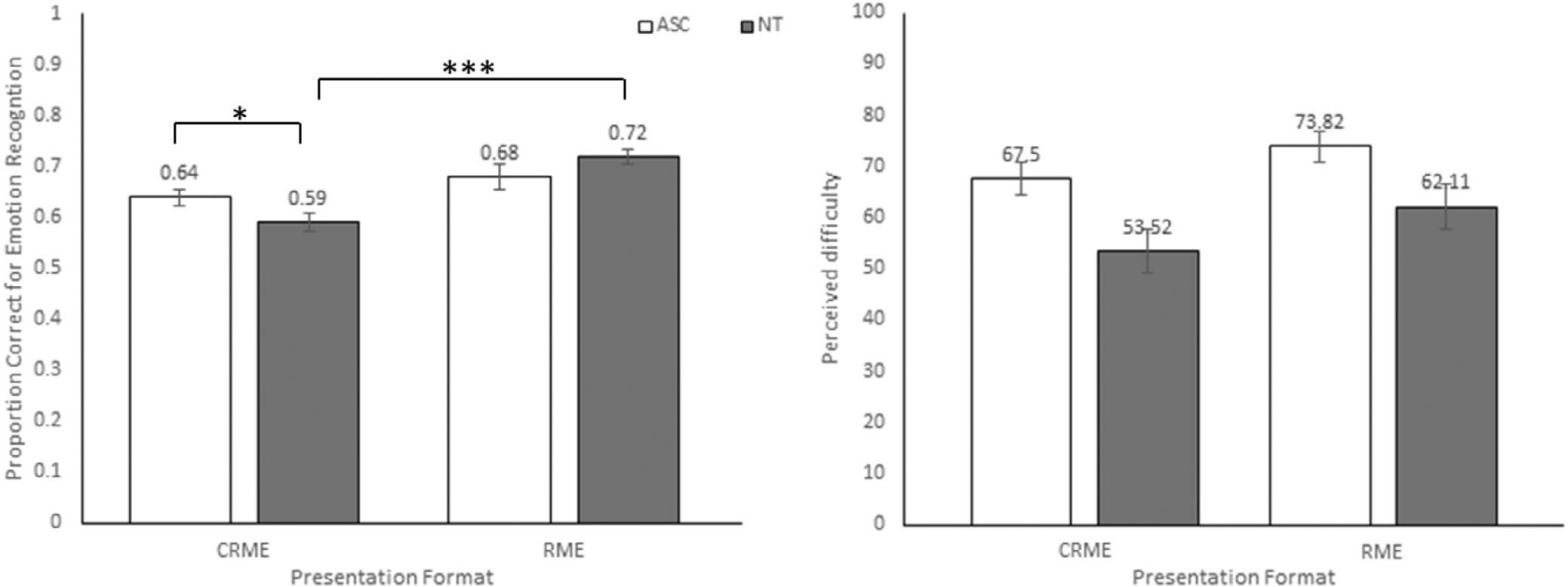
Mean and standard errors for facial emotion recognition C/Reading the Mind in the Eyes proportion correct scores (left panel) and perceived difficulty (right panel) divided by ASCs and NTs.

There was no main effect of either Presentation format *F*(1,186) = 4.586, *p* = 0.278, *η* ^2^ = 0.821 or Diagnosis *F*(1,186) = 0.094, *p* = 0.811, *η* ^2^ = 0.086. However, the interaction between Diagnosis and Presentation format was significant *F*(1,186) = 4.919, *p* = 0.028, *η* ^2^ = 0.026. To explore the interaction further, two independent‐sample t‐tests were conducted, investigating the difference between ASC and NT groups in the RME and C‐RME separately. The proportion of correct emotion recognition was not significantly different between ASCs and NTs in the RME condition (*t*[79.44] = 1.03, *p* = 0.31, *d* = 0.21). In contrast, ASCs scored higher than NTs in the C‐RME condition (*t*[92] = 2.25, *p* = 0.027, *d* = 0.47). We also explored the interaction by conducting two independent‐sample *t* tests investigating whether the ASCs' (and NTs') performance differed between the RME and C‐RME conditions. ASCs' performance was similar in the two conditions (*t*[84.84] = 1.64, *p* = 0.11, *d* = 0.33). In contrast, NTs' performance was higher in the RME condition compared to the C‐RME condition (*t*[92] = 5.59, *p <* 0.001, *d* = 1.17). As also demonstrated in Figure 2 (left panel), this interaction showed that while NTs' and ASCs' performance was similar when presented with human photographs, autistic individuals outperformed NTs when they saw cartoons. This seems to be due to the fact that the performance of autistic individuals was not affected by the stimulus presented. Meanwhile, control individuals performed worse when they had to judge cartoons rather than pictures.

Interestingly, ratings of perceived difficulty did not follow this pattern (see Figure 2, right panel). A univariate ANOVA with task difficulty as the dependent variable showed that the main effects of Presentation format *F*(1,186) = 43.13, *p* = 0.096, *η* ^2^ = 0.997 and Diagnosis *F*(1,186) = 128.03, *p* = 0.056, *η* ^2^ = 0.992 were not significant and neither was the interaction between Diagnosis and Presentation format, *F*(1,186) = 0.109, *p* = 0.742, *η* ^2^ = 0.001. See Table 2 for means and SDs. Correlation analyses investigating the relationship between perceived difficulty and task performance can be found in the Appendix S1.

**TABLE 2:** Mean and standard deviations for average facial emotion recognition difficulty scores.

		Mean	SD
RME	ASC	73.82	21.18
	NT	62.11	20.91
C‐RME	ASC	67.5	24.07
	NT	53.52	27.67

## DISCUSSION

Results showed that autistic people did not perform significantly worse on the RME than NTs. While this was not as hypothesized, it may be that the high proportion of female participants boosted ASC performance. Previous work, for instance, has shown that autistic females do not perform differently to NT females (Holt et al., 2014) and that there is a female advantage in the RME (Kirkland et al., 2013). Additionally, this may be explained by adding definitions to the RME to remove variance associated with vocabulary differences. In line with our predictions, individuals with ASC outperformed NTs on the cartoon version of the task. These results suggest that autistic individuals may lack a human‐specific specialization in this domain seen in NTs. For instance, NTs performed better on the RME than the C‐RME, while this was not the case for autistic individuals, whose performance on the two versions did not significantly differ. This is the first study to find that autistic people outperform NTs on the RME.^1^ Our results suggest that NT people have more difficulty than autistic people when the agent being evaluated is anthropomorphic. Our findings also suggest that autistic people are perhaps not experiencing the same processing deficits as NTs when taking the C‐RME. Why might this be?

We speculated that this could be driven by either a reduced motivation for actual human agents or an increased motivation to evaluate cartoon agents. The descriptive statistics in the present study suggest that the interaction seen in the present study was likely driven not by autistic individuals over performing on the C‐RME but by NTs underperforming on the C‐RME. Additionally, descriptive statistics relating to the measure of difficulty suggested that the autistic individuals found both tasks more difficult than NTs on average. However, this difference was not significant at the 0.05 level.

If NTs were to be used as a ‘benchmark’ for FER development, it would appear that there is a specialization for the human in typical development. As a result, anthropomorphic FER is more difficult for NTs. Considering that autistic people do not see a reduced performance on such measures, they may not experience the same specialized interest or aptitude for human FER. Interestingly, this does not lead to the deterioration of anthropomorphic FER. Specifically, autistic performance was not lower across both conditions, and it did not follow the same pattern as NTs. As such, it may be that autistic people also have an enhanced ability to perform anthropomorphic FER. This ability may develop through protracted engagement with anthropomorphic agents, as is suggested through research on restricted interests. The enhanced development of anthropomorphic FER in autistic people would contrast with research suggesting that FER deficits only increase as autistic people age (Lozier et al., 2014). Instead, perhaps autistic people, and those with high autistic traits, continue to develop anthropomorphic FER and ToM which allows them to eventually surpass NTs. This continued development may explain why studies on very young autistic children show impaired performance in both human and nonhuman face recognition (Chawarska & Volkmar, 2007), while studies on older autistic children, adolescents and adults show an intact or even relatively enhanced ability for nonhuman performance (for a review, see Atherton & Cross, 2018).

Autism research is rife with studies showing that autistic people enjoy engaging with the nonhuman and may be doing so increasingly throughout development, whether it be through animation (Holmgaard et al., 2013), contact with pets (Atherton et al., 2022), animal‐assisted therapy (O'Haire, 2013), or even embodying the nonhuman during online game‐play (Stendal & Balandin, 2015) (for a review, see Atherton & Cross, 2018). This type of engagement may allow autistic people to develop social expertise and derive social pleasure in ways that do not rely on human specialization, which may function as an extension of how autistic people begin to see themselves as more than human (Davidson & Smith, 2009). Future research should look to better understand autistic people's motivations for interacting with anthropomorphic agents. Cross et al. (2019) and Carter et al. (2016) suggest that using anthropomorphic agents in therapeutic contexts may also improve social understanding and connection. Including such agents in virtual settings and observing changes in responsiveness would be a valuable avenue for future research.

## CONFLICT OF INTEREST

The authors have no conflicts of interest to declare. All co‐authors have seen and agree with the manuscript's contents, and there is no financial interest to report. We certify that the submission is original work and is not under review at any other publication.

## Supporting information


**Appendix S1:** Supporting Information

## Data Availability

The data that support the findings of this study are available from the corresponding author upon reasonable request.

## Footnotes

^1^
It should be noted that the ANOVA interaction and main effects are not significant if the six outliers are included in the analysis (main effect of Format *F*(1,192) = 24.611, *p* = 0.127, η^2^ = 0.961; main effect of Diagnosis *F*(1,192) = 1.632, *p* = 0.423, η^2^ = 0.620; interaction *F*(1,192) = 0.613, *p* = 0.435, η^2^ = 0.003). This would suggest that the performance was equal across the four experimental conditions when low scoring outliers were not removed.
