# Supplementary material for: Autistic people outperform neurotypicals in a cartoon version of the Reading the Mind in the Eyes
Source: Autism Res. 2022 Jul 20;15(9):1603–8. doi: 10.1002/aur.2782 (PMC9543219; doi:10.1002/aur.2782)
Supplement: Supplementary file 1 — Appendix S1: Supporting Information [file AUR-15-1603-s001.docx]

**Supplementary Materials**


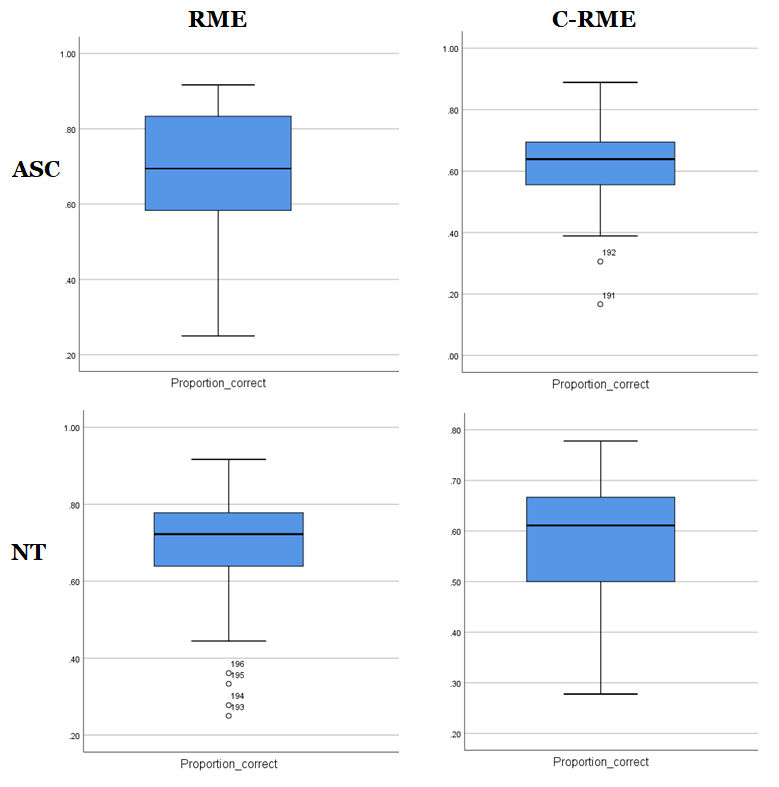


Figure A. Boxplots of the proportion of correct responses in each experimental condition. These boxplots were used to determine outliers with poor performance. Participants were classified as outliers if their performance was below the first quartile minus 1.5 times the interquartile range or above the third quartile plus 1.5 times the interquartile range.

Pearson’s correlations were conducted to test the relationship between the perceived difficulty of the task and the proportion of correct responses made by participants. The relationship failed to be significant when all participants (ASCs and NTs) and conditions (RME and C-RME) were included in the analysis (r= -.14, p= .063). Given that the p-value was close to significance, the correlation analysis was repeated for each group and condition combination. NTs’ performance in the RME task (r= -.11, p= .491) and in the C-RME task (r= -.24, p= .096) did not correlate with their perception of task difficulty. ASCs’ performance in the C-RME task also did not correlate with their perception of task difficulty (r= -.22, p= .136). However, ASCs’ performance in the RME task significantly correlated with how difficult the task was perceived (r= -.31, p= .030). In other words, the more difficult the RME task with human faces was perceived by individuals with autism, the poorer their performance. This could suggest that ASCs were particularly conscious of their difficulty in performing the task.
